# Biallelic *SLCO2A1* variants in two siblings with primary hypertrophic osteoarthropathy and possible chronic enteropathy

**DOI:** 10.3389/fendo.2026.1783445

**Published:** 2026-09-10

**Authors:** Tao Wang, Qian Zhang, Tingting Shi, Xin Chen, Li Wang, Xiuming Liu, Yihui Wang, Kaikai Shen, Fei Zhao, Litao Qin

**Affiliations:** 1 Medical Genetic Institute of Henan Province, Henan Provincial People’s Hospital, People’s Hospital of Zhengzhou University, People’s Hospital of Henan University, Zhengzhou, China; 2 Department of Ultrasound, Henan Provincial People’s Hospital, People’s Hospital of Zhengzhou University, People’s Hospital of Henan University, Zhengzhou, China; 3 Department of Pharmacy, Henan Provincial People’s Hospital, People’s Hospital of Zhengzhou University, People’s Hospital of Henan University, Zhengzhou, China

**Keywords:** biallelic variants, chronic enteropathy associated with SLCO2A1 gene, genotype-phenotype correlation, primary hypertrophic osteoarthropathy, SLCO2A1

## Abstract

**Background and aims:**

Pathogenic variants in the *SLCO2A1* gene are responsible for two rare monogenic disorders: primary hypertrophic osteoarthropathy (PHO) and chronic enteropathy (CEAS). The clinical and genetic characteristics of individuals with either PHO or CEAS have been extensively documented in previous studies. However, the clinical and genetic features of patients presenting with both PHO and CEAS concurrently remain largely unknown. This study aims to elucidate the clinical and genetic features of patients with overlapping PHO and CEAS.

**Methods:**

We evaluated two affected individuals from the same family presenting with both PHO and possible CEAS. Next, a systematic literature review was conducted across the PUBMED, EMBASE, and China National Knowledge Infrastructure (CNKI) databases, covering the period from January 1, 2000, to November 30, 2025. Clinical and genetic data from the literature cohort and our index were collected and analyzed.

**Results:**

Two novel compound heterozygous variants (NM_005630.3: c.232G>T and c.1929C>G) in the *SLCO2A1* gene were identified in our two familial patients. In the broader cohort (n=54), the disease demonstrated prominent sexual dimorphism. Males accounted for 75.9% (41/54) of the cohort and typically presented with classic PHO phenotypes, including cutis gyrata, pachydermia, digital clubbing, periosteal thickening, and arthralgia. In contrast, female patients primarily presented with gastrointestinal manifestations, such as ulcers, abdominal pain, obstruction, diarrhea, mucosal abnormality, and bleeding. Genetic analysis revealed that the variant allele frequencies of NM_005630.3: c.940+1G>A and c.1807C>T were 25.9% and 25%, respectively, across the entire cohort.

**Conclusions:**

This study identified two novel compound heterozygous variants (NM_005630.3: c.232G>T and c.1929C>G) in *SLCO2A1*, thereby expanding the variant spectrum of *SLCO2A1*. Furthermore, our systematic analysis identifies distinct, sex-biased clinical phenotypes in patients with concurrent PHO and CEAS. Based on these findings, we propose a gender-specific clinical consideration to enhance the identification and management of *SLCO2A1*-associated disorders.

## 1 Introduction

Variants in the *SLCO2A1* gene cause two rare monogenic disorders: primary hypertrophic osteoarthropathy (PHO) and chronic enteropathy (CEAS). PHO is an autosomal recessive disease that affects the skin and skeletal system; it typically presents with digital clubbing, periostosis, acroosteolysis, painful joint enlargement, and thickened skin (1–3). Similarly, CEAS is an autosomal recessive disorder characterized by multiple small intestinal ulcers, chronic anemia, and hypoproteinemia (4, 5). There is a significant gender disparity between PHO and CEAS. PHO predominantly affects males, while CEAS affects a higher proportion of females (6, 7). Recent studies have indicated that patients harboring *SLCO2A1* variants can present with an overlapping phenotype of both PHO and CEAS, with a distinct predominance observed in male patients (5, 8, 9). However, the clinical characteristics of patients with overlapping PHO and CEAS remain largely unknown.

Prostaglandins (PGs) are synthesized from arachidonic acid by two distinct isoforms of cyclooxygenase (COX-1 and COX-2). Among these, PGE_2_ produced by COX-2 is the most potent inflammatory mediator (10). Current evidence suggests that the *SLCO2A1* gene encodes a prostaglandin transporter (PGT), which transports PGs into cells and plays a vital role in regulating PGs inactivation and distribution (6, 11). A deficiency in the *SLCO2A1* gene disrupts the transport of PGE_2_ from the extracellular to the intracellular environment, resulting in impaired PGE_2_ degradation (12). Consequently, individuals with *SLCO2A1* gene deficiency exhibit increased extracellular levels of PGE_2_, which bind to PG receptors on the cell surface, leading to sustained activation of the second messenger cyclic adenosine monophosphate (cAMP) (6). Although the mechanism linking *SLCO2A1* deficiency to CEAS remains to be fully elucidated, studies using an *Slco2a1*-deficient mouse model of colitis suggest that activation of NLRP3 inflammasomes in macrophages contributes to severe intestinal inflammation (13).

To date, more than 100 pathogenic variants have been identified in patients with PHO or CEAS, exhibiting a range of pathogenic effects (6, 8). Among these, the most common variant is the splice site variant NM_005630.3: c.940 + 1G>A, which results in exon 7 skipping (14). Besides, various studies have reported other pathogenic variants, including missense, nonsense and frameshift variants. However, a comprehensive analysis of the genetic features of *SLCO2A1* variants in patients with both PHO and CEAS remains limited.

We report two novel compound heterozygous *SLCO2A1* variants in a pedigree of two male patients with concurrent PHO and possible CEAS. By analyzing these cases alongside a literature review and genotype-phenotype analysis, we aim to delineate previously unreported clinical and genetic features of this dual presentation.

## 2 Materials and methods

### 2.1 Patient and samples

This study involved two affected individuals from the same pedigree: a 15-year-old pubertal male and his older brother, who was diagnosed with PHO at the age of 18. Written informed consent was obtained from all family members participating in the study prior to enrollment. This study was conducted in strict adherence to the Declaration of Helsinki and received ethical approval from the Ethics Committee for Clinical Trials of Drugs (Devices) of Henan Provincial People’s Hospital (Ethical Approval No. 2024-090). Peripheral blood samples (2 mL each) were collected from the proband and related family members using EDTA-anticoagulated tubes.

### 2.2 Genomic DNA extraction

Genomic DNA was extracted from the peripheral blood lymphocytes of all family members using a genomic DNA isolation kit (Bioteke, Beijing, China) according to the manufacturer’s standard protocol. The concentration and purity of the extracted DNA were assessed using a NanoDrop2000 spectrophotometer, and the DNA was stored at -20°C for subsequent experiments.

### 2.3 Whole exome sequencing

Whole exome sequencing (WES) was performed according to the previously reported methods (15, 16). Briefly, a total of 50 ng of DNA underwent fragmentation, followed by end-repair, addition of a 3’dA overhang, and adapter ligation. Then, purified DNA fragments were hybridized and captured by the NanoWES kit 1.0 (Berry Genomics, Beijing, China). Finally, the exome library was quantified by qPCR and sequenced on an Illumina NovaSeq 6000 (Illumina, CA, USA) with 150-base paired-end reads.

### 2.4 Bioinformatics analysis

The subsequent analyses of the WES data were conducted in accordance with the guidelines for NGS bioinformatics pipelines (17). Raw data were aligned to the human reference genome (GRCh37) using BWA, and duplicates were removed using Picard v2.10.7. GATK and ANNOVAR were employed for variant calling and variant annotation. The filtration process excluded variants with a minor allele frequency (MAF) exceeding 0.01 in the ExAC (Exome Aggregation Consortium), gnomAD (Genome Aggregation Database), and 1000 Genomes Project. A variant was considered novel if not reported at the time of data collection.

The candidate pathogenic variations were validated by Sanger sequencing. Conservation analysis of amino acids was conducted across different species utilizing UGENE software. In silico prediction algorithms (SIFT and Polyphen-2) were employed to assess the variant's function. All suspected variants were classified according to the guidelines of the American College of Medical Genetics and Genomics (ACMG).

Furthermore, the impact of the candidate pathogenic variant on the protein structure was assessed through three-dimensional protein modeling using the SWISS-MODEL online platform (https://swissmodel.expasy.org/). The FASTA sequence of the protein *SLCO2A1* (NP_005621.2) was submitted to the SWISS-MODEL automated server for homology modeling, resulting in the identification of three potential models, designated as Model-1, Model-2, and Model-3. Among these, Model-2, which utilized a template from AlphaFold v2.0, was selected for further analysis due to its superior attributes, including a sequence identity of 100%, complete sequence coverage (1.00), and the highest Global Model Quality Estimation (GMQE) score of 0.82.

To analyze the distribution of mutation-induced amino acid alterations within the secondary structure of *SLCO2A1*, the protein's membrane topology was determined. The reference sequence and topological domain information for *SLCO2A1* were obtained from the UniProtKB database under accession number Q92959 (https://www.uniprot.org/uniprotkb/Q92959/entry#ptm_processing). Subsequently, the amino acid changes were manually annotated onto the protein structure using Adobe Illustrator CS (v11.0) graphics software.

### 2.5 Search strategy and selection criteria

**Figure 1:**
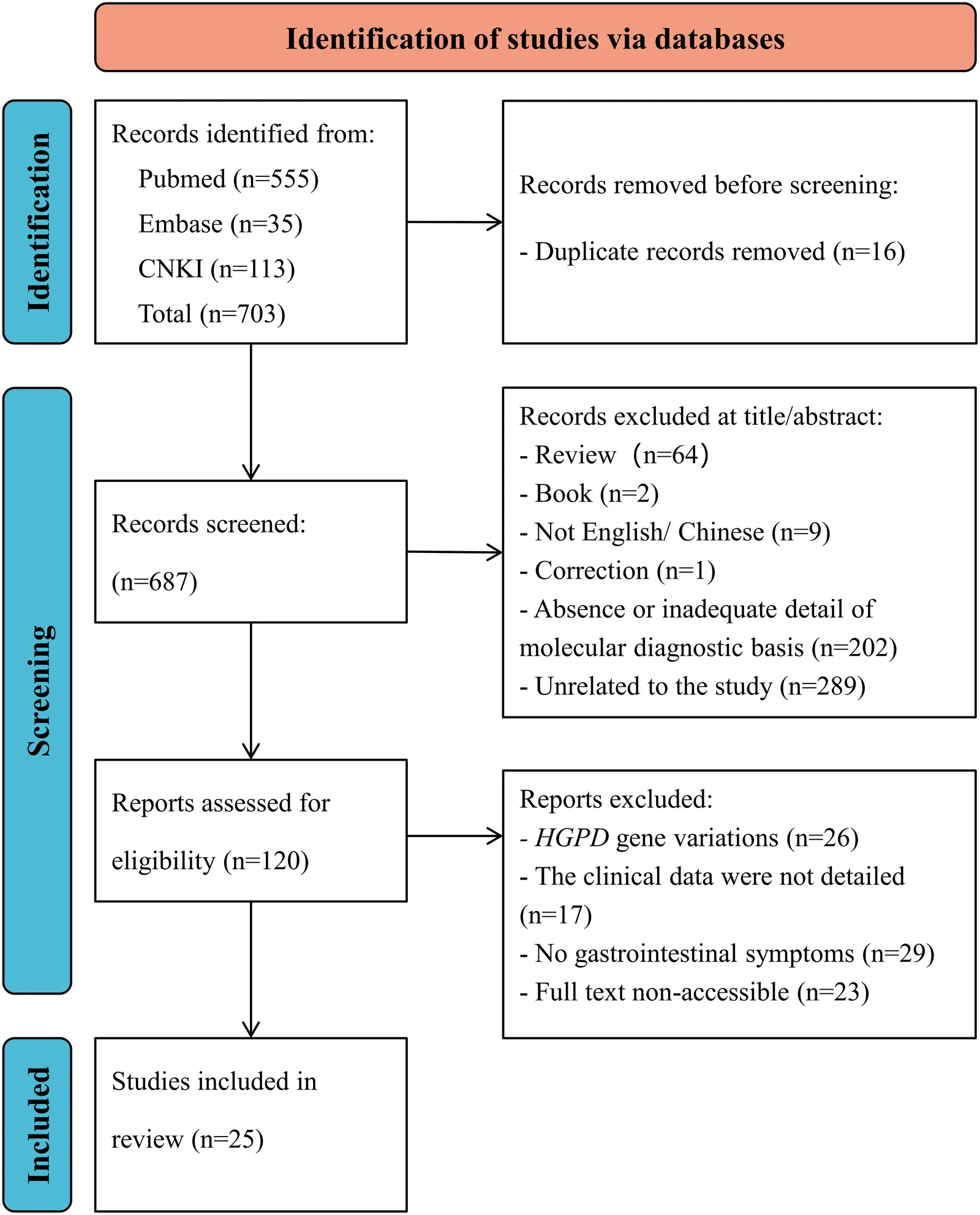
Flowchart of the inclusion of studies for analysis of the clinical and genetic features in patients with both PHO and CEAS. CNKI, China National Knowledge Infrastructure databases; PHO, primary hypertrophic osteoarthropathy; CEAS, chronic enteropathy associated with the SLCO2A1 gene.

This study conducted a systematic literature search across the PUBMED, EMBASE, and China National Knowledge Infrastructure (CNKI) databases, covering the period from January 1, 2000, to November 30, 2025. Given the more inclusive indexing of the EMBASE database, a rigorous search strategy was employed, incorporating Emtree terms, word variants, and keywords in the following query: “(*SLCO2A1*) AND (Chronic enteropathy OR primary ulcer) AND (Primary hypertrophic osteoarthropathy OR pachydermoperiostosis OR Touraine-Solente-Gole syndrome)”. Conversely, to capture potentially relevant reports in the PUBMED database, the search strategy utilized combinations of relevant Medical Subject Headings (MeSH) terms, word variants, and keywords. This approach specifically targeted the query: “(*SLCO2A1*) AND (Chronic enteropathy) OR (primary ulcer) AND (Primary hypertrophic osteoarthropathy) OR (pachydermoperiostosis) OR (Touraine-Solente-Gole syndrome)”. For the CNKI database, the search was conducted using the keywords: (*SLCO2A1*) OR (primary hypertrophic osteoarthropathy) OR (pachydermoperiostosis) OR (Touraine-Solente-Gole syndrome). Besides, bibliographies of high-impact articles were reviewed to identify additional relevant studies, which were included in our references when appropriate. The processes of literature screening and data extraction were conducted independently by two investigators. Disagreements were resolved through discussion; if a consensus could not be reached, a final decision was made by a third senior investigator. To prevent the inclusion of duplicate patients or overlapping cohorts, duplicate records identified through database searches were eliminated. Baseline patient information, clinical phenotypes, and genetic variant data from various publications within the included studies were manually verified. Patients exhibiting completely identical core information were included only once to ensure data independence and prevent duplication. Articles included in this study were required to meet the criteria as follows (**Figure 1**): (1) patients were diagnosed with the clinical manifestations of CEAS, and (2) the study contained information about genetic testing results of the *SLCO2A1* gene. Reviews, books, duplicate records, and publications not in English or Chinese were excluded.

## 3 Results

### 3.1 Clinical features

**Figure 2:**
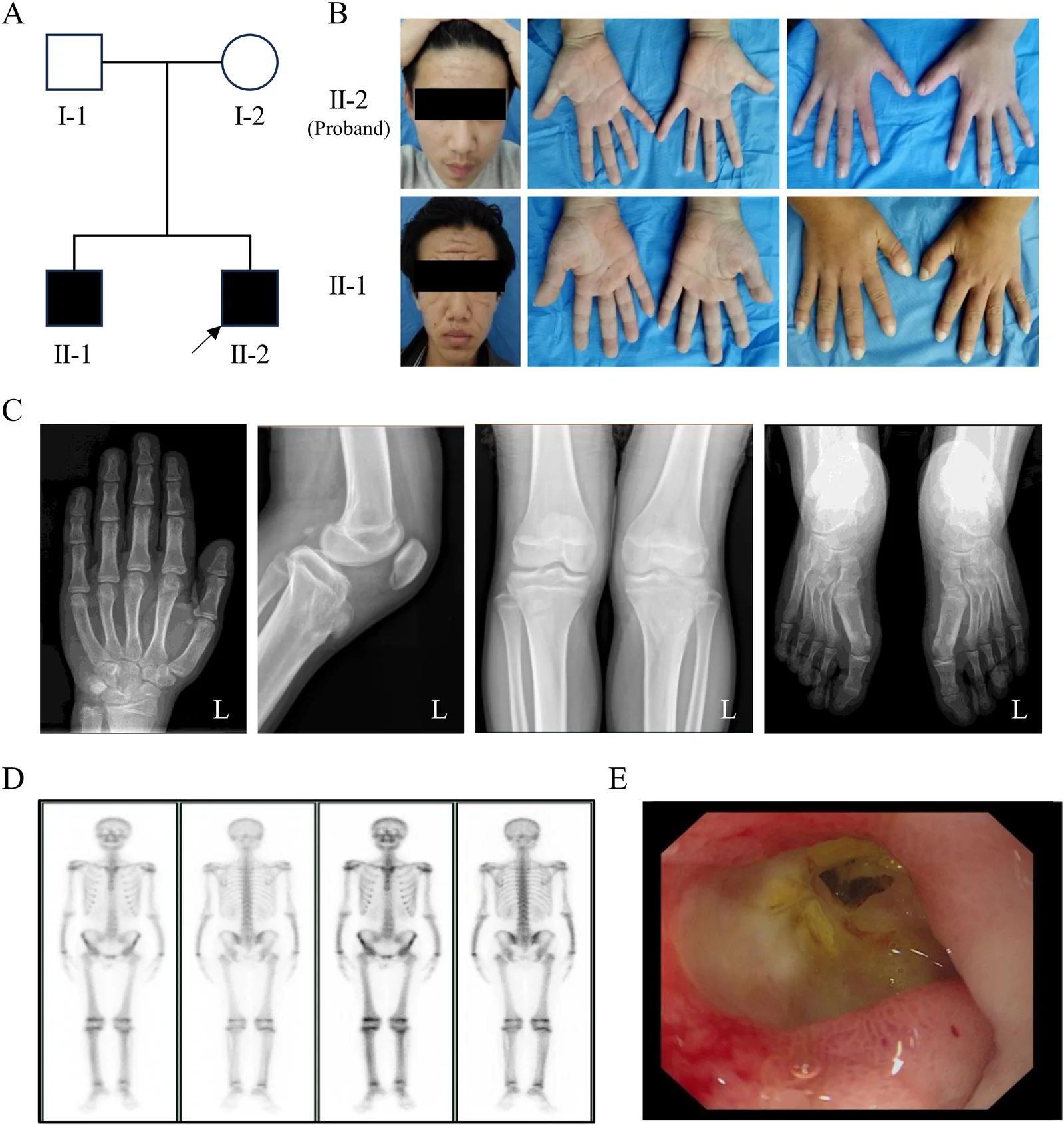
Clinical manifestations of the patients **(A)** The pedigree in this study; **(B)** Cutis gyrata, pachydermia and digital clubbing in II-1 and II-2; **(C)** Imaging studies revealed abnormalities in the cortex of the radial side bone, associated tissue swelling, and the presence of a valgus deformity; **(D)** Whole-body bone scintigraphy demonstrated clearly defined skeletal structures; **(E)** Gastroscopy revealed a duodenal bulb ulcer with resultant stenosis. The black arrows indicate the proband. Squares and circles indicate males and females, respectively. L, left side.

The pedigree comprised phenotypically normal parents and two affected offspring (**Figure 2A**). The 15-year-old male proband (II-2) presented with a one-month history of progressive pachydermia on the forehead, characterized by pronounced furrowing and altered facial features (**Figure 2B**). The skin on his hands, feet, and ankles was notably thickened, with swelling of the digital, knee, and ankle joints. He also reported knee pain that worsened with squatting and standing.

The patient’s clinical and laboratory findings are summarized in **Table 1**. Imaging studies revealed an abnormality in the radial bone cortex of the middle phalanx of the left middle finger, a flaky bone density shadow at the left tibial tubercle, and adjacent soft tissue swelling near the left tibial tubercle. Besides, the tibial joint space of the left knee was narrower than that of the right, accompanied by a valgus deformity of both halluces (**Figure 2C**). Whole-body bone scintigraphy revealed a symmetrical skeletal framework characterized by symmetrically elevated radiotracer uptake within the skull, facial bones, and appendicular long bones. (**Figure 2D**). Based on these findings, a clinical diagnosis of PHO was strongly suspected.

**Table 1:** Laboratory data for the proband of PHO.

Parameter	Result	Reference range
Hemoglobin (g/L)	124↓	130~175
CRP (mg/L)	11.61↑	0~10
ESR (mm/h)	31↑	0~20
IGFI (ng/ml)	217↓	237~996
Ro-52 (WB)	+	–
Osteocalcin (ng/ml)	193.8↑	24~70
PINP (ng/ml)	678.3↑	16.89~65.49
β-CrossLaps	3.43↑	0
25-(OH)V (ng/ml)	9.45↓	≥20

CRP, C-reactive protein quantification; ESR, Erythrocyte sedimentation rate; IGFI, Insulin-like growth factor I; PINP, Procollagen I N-Terminal Peptide; ↑, higher than the normal reference range; ↓, below the normal reference range; +, positive; -, negative.

The proband’s brother (II-1) exhibited clinical manifestations similar to those observed in the proband. Dermatological examination revealed marked thickening and coarseness of the forehead skin, presenting as a classic cerebriform pattern (cutis verticis gyrata), accompanied by prominent digital clubbing of the fingers (**Figure 2B**). Besides, he experienced recurrent abdominal pain over the past four years. A gastroscopy revealed a duodenal bulb ulcer with resultant stenosis (**Figure 2E**), while the abdominal CT scan showed no significant abnormalities. He was also diagnosed with mild hypoproteinemia and a positive fecal occult blood test. However, the patient refused to undergo a colonoscopy. Characteristic ulcerative lesions in the small intestine, along with histopathological analysis of biopsy specimens, are pivotal indicators of gastrointestinal lesions as per the diagnostic criteria for Chronic Enteropathy Associated with *SLCO2A1* disease (CEAS) (5). Consequently, due to inadequate evidence of gastrointestinal lesions, the siblings were cautiously diagnosed with possible CEAS.

### 3.2 Identification of the compound heterozygous variants in the *SLCO2A1* gene

**Figure 3:**
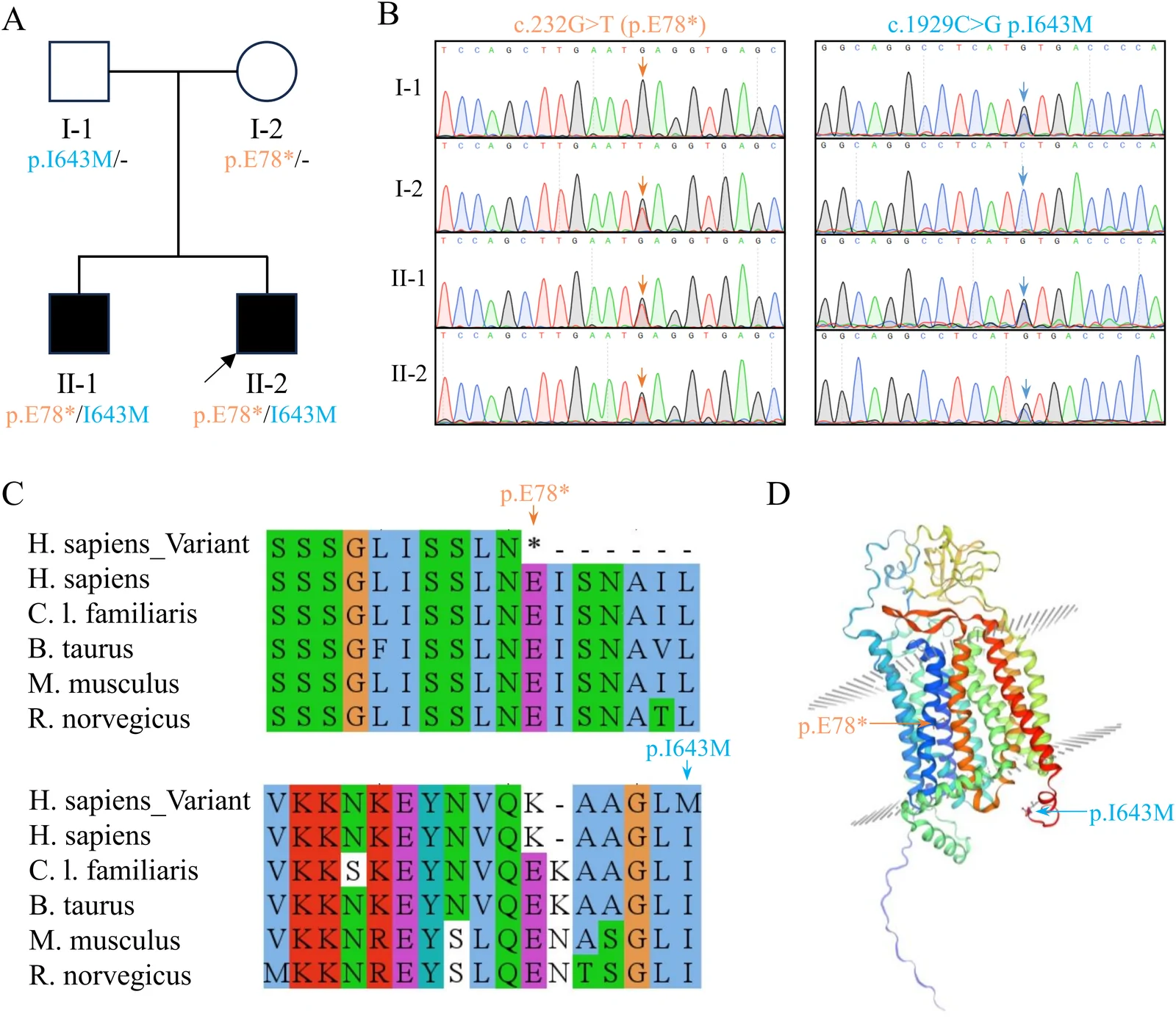
Genetics and *in silico* analysis of NM_005630.3: c.232G>T and c.1929C>G **(A)** The pattern of variants observed in the pedigree analyzed in this study; **(B)** Sanger sequencing results for family members of the pedigree; **(C)** Conservation analysis on the mutated amino acid site across various mammals; **(D)** Structural localization of *SLCO2A1* variants. The black arrows indicate the proband. Squares and circles indicate males and females, respectively. The orange and blue arrows indicate the locations of the c.232G>T (p.E78*) and c.1929C>G (p.I643M) variants, respectively, in the Sanger sequencing, conservation analysis, and three‑dimensional protein model. I, isoleucine; M, methionine; E, glutamic acid; *, termination.

Two variants in the *SLCO2A1* gene, a nonsense variant (NM_005630.3: c.232G>T (p.Glu78Ter)) and a missense variant (NM_005630.3: c.1929C>G (p.Ile643Met)), were identified in the proband (II-2) using whole exome sequencing. Sanger sequencing confirmed that these variants were inherited from the proband’s parents: the mother (I-2) carried the c.232G > T (p.Glu78Ter) variant, and the father (I-1) carried the c.1929C > G (p.Ile643Met) variant. Moreover, the proband’s brother (II-1) carried both variants (**Figures 3A, B**). Overall, these findings indicated that the two compound heterozygous variants are candidate pathogenic variants within the *SLCO2A1* gene responsible for the disease phenotype in this pedigree.

### 3.3 Genetic analysis

The NM_005630.3: c.232G>T (p.Glu78Ter) variant, located in exon 2 of the *SLCO2A1* gene, resulted in a nonsense variant at amino acid position 78 of SLCO2A1, leading to a premature stop codon that potentially causes a complete loss of gene function. This variant was absent from all major public genomic databases (gnomAD, ExAC, and the 1000 Genomes Project) as well as from variant repositories such as ClinVar and HGMD. *In silico* prediction algorithms classified this variant as pathogenic. Conservation analysis across multiple species revealed that amino acids in the C-terminus were highly conserved (**Figure 3C**). Furthermore, the three-dimensional protein model illustrated the location of the variant p.Glu78Ter (**Figure 3D**). Building upon the above findings, the NM_005630.3: c.232G>T (p.Glu78Ter) variant was classified as pathogenic according to the ACMG criteria, with evidence levels: PVS1+PM2_Supporting+PP1+PP4 (**Supplementary Table 1**).

The NM_005630.3: c.1929C>G (p.Ile643Met) variant, located in exon 14 of the *SLCO2A1* gene, results in the substitution of the hydrophobic amino acid isoleucine (Ile) with another hydrophobic, non-polar amino acid, methionine (Met), at codon 643 of the protein, introducing a more flexible and unbranched side chain. The variant found has hitherto not been described in gnomAD, ExAC, the 1000 Genomes Project, or variant databases such as ClinVar and HGMD. The variant was predicted to be “possibly damaging” with a score of 0.930 by PolyPhen-2 and “damaging” with a score of 0.040 by SIFT. Conservation analysis in multiple species showed that p.Ile643Met was located in a highly conserved region (**Figure 3C**), and its spatial location was further visualized via the three‑dimensional protein model (**Figure 3D**). According to ACMG criteria, this variant was classified as likely pathogenic: PM2_Supporting + PM3 + PP1 + PP3 + PP4 (**Supplementary Table 1**).

### 3.4 Treatment

Based on clinical manifestations and genetic testing data, the siblings were diagnosed with PHO and possible CEAS. During hospitalization, the patient (II-1) received omeprazole (40 mg/day) and methylprednisolone (40 mg/day) for 5 days to mitigate gastrointestinal discomfort and inflammation. Simultaneously, colchicine (1 mg/day) and diclofenac (75 mg/day) were administered to alleviate joint pain. Besides, oral iron (300 mg/day) and calcium carbonate (600 mg/day) were prescribed to address anemia and prevent osteoporosis, respectively. Following discharge, the patient was maintained on colchicine, diclofenac, iron, and calcium carbonate. After three months, marked resolution of the facial pachydermia was observed, and the patient’s upper abdominal pain had subsided. C-reactive protein levels decreased from 34.4 mg/L to 24.1 mg/L, and hemoglobin levels increased from 103 g/L to 118 g/L over the course of one year. Nevertheless, despite the amelioration of PHO symptoms, the patient continued to experience hypoalbuminemia and mild anemia.

Upon the diagnosis of PHO and possible CEAS in the proband (II-2), a combination therapy regimen was implemented, comprising oral etoricoxib (60 mg/day), colchicine (1 mg/day), and topical isotretinoin. After six months of follow-up, this treatment strategy resulted in a notable reduction in facial skin induration and a significant alleviation of knee swelling.

### 3.5 Clinical characteristics of patients diagnosed with both PHO and CEAS

CEAS is another rare monogenic disorder caused by *SLCO2A1* defects. The primary characteristic of CEAS is the presence of multiple intestinal ulcers. However, the clinical and genetic characteristics of CEAS remain largely unknown, particularly in patients who also present with PHO. Herein, we summarized the genetic associations and clinical characteristics of patients with both CEAS and PHO to provide a comprehensive understanding of *SLCO2A1-*associated disorders.

From an initial pool of 703 publications, 25 studies were ultimately selected for full-text review after meeting the established inclusion criteria for analysis (**Figure 1** and **Supplementary Table 3**). A total of 54 patients were enrolled in this study, comprising 41 males and 13 females, with a median age of onset of 16 years for both genders (**Table 2** and **Supplementary Table 2**). Clinical manifestations associated with PHO were predominantly observed in male patients, including *cutis gyrata* (100% in males, 0% in females), pachydermia (100% in males, 7.69% in females), digital clubbing (100% in males, 30.77% in females), and arthralgia (97.06% in males, 7.69% in females). Notably, periosteal thickening was present in 100% of male and female patients, suggesting its potential diagnostic significance in females.

**Table 2:** Comparison of clinical finding of PHO & CEAS patients by sex.

Clinical Features	Male(n=41)	Female(n=13)	p*
Age at onset (years, median)	16	16	NS
Gastrointestinal symptoms			
Abdominal pain	4(9.76%, n=41)	12(92.31%, n=13)	**<0.0001**
Ulcers	20(48.78%, n=41)	13(100%, n=13)	**0.00034**
Diarrhea	15(36.59%, n=41)	1(7.69%, n=13)	**0.024**
Obstruction	10(24.39%, n=41)	7(53.85%, n=13)	NS
Bleeding	12(29.27%, n=41)	1(7.69%, n=13)	NS
Mucosal abnormality	15(36.59%, n=41)	1(7.69%, n=13)	**0.042**
Other(Polyp/Inflammation)	7(17.07%, n=41)	0(0%, n=13)	NS
Disease site			
Stomach	28(77.78%, n=36)	2(15.38%, n=13)	**0.0002**
Duodenum	9(25.00%, n=36)	3(23.08%, n=13)	NS
Jejunum	9(25.00%, n=36)	4(30.77%, n=13)	NS
Ileum	16(44.44%, n=36)	13(100%, n=13)	**<0.0001**
Colon	1(2.78%, n=36)	3(23.08%, n=13)	**0.0259**
Extra-intestinal manifestations			
Cutis gyrata	28(100%, n=28)	0(0%, n=12)	**<0.0001**
Pachydermia	41(100%, n=41)	1(7.69%, n=13)	**<0.0001**
Digital clubbing	41(100%, n=41)	4(30.77%, n=13)	**<0.0001**
Periosteal thickening	39(100%, n=39)	3(100%, n=3)	NS
Arthralgia	33(97.06%, n=34)	1(7.69%, n=13)	**<0.0001**

NS, not significant; Significant p values are indicated in bold; *: Fisher’s exact test or Mann-Whitney U test.

Gastrointestinal symptoms were prevalent among all patients, with ulcers the most frequently observed, affecting 48.78% of male patients and 100% of female patients. In male patients, the symptoms following ulcers in frequency were diarrhea and mucosal abnormality, each reported in 36.59% of cases, whereas abdominal pain was the second most common symptom in female patients, occurring in 92.31% of cases. Obstruction was noted in 24.39% of male patients and 53.85% of female patients, indicating a significantly higher incidence in females. Moreover, bleeding and abdominal pain were observed in 29.27% and 9.76% of male patients, respectively. The ileum was the primary site of affliction in female patients (100%), while in male patients, the stomach was predominantly affected (77.78%). Other affected sites in males included the ileum (44.44%), jejunum (25%), and duodenum (25%), whereas in females, the jejunum (30.77%), duodenum (23.08%), and colon (23.08%) were also involved. The observed differences in predominant clinical symptoms between male and female patients may be attributed to sex-specific factors influencing the initial diagnosis. Male patients primarily exhibited features of PHO, with gastrointestinal diagnoses being secondary. In contrast, female patients generally presented with more severe gastrointestinal symptoms as their primary complaint. The distinction between male and female patients is likely due to *SLCO2A1* expression being influenced by modifiers such as sex-influenced genes or related hormones.

### 3.6 Genetic features of *SLCO2A1* variants causing PHO and CEAS

Molecular genetic analysis revealed that all patients carried biallelic variants in the *SLCO2A1* gene. Among the 54 patients, 29 genetic variants were identified. Notably, all 29 variants were present in male patients, whereas only 3 were detected in female patients (**Table 3**). The types of variants identified included missense variants (n=17, 58.6%), frameshift variants (n=5, 17.2%), splice-site variants (n=3, 10.3%), nonsense variants (n=3, 10.3%), and synonymous variants (n=1, 3.4%). The allele frequencies of NM_005630.3: c.940 + 1G>A (p.Arg288GlyfsTer7) and c.1807C>T (p.Arg603Ter) among the 54 patients included in this study were 25.9% and 25%, respectively. Notably, among the 13 female patients, the allele frequencies for these two variants were 38.46% and 53.85%, respectively. These findings collectively suggest that the NM_005630.3: c.940 + 1 G>A and c.1807C>T variants possess significant molecular diagnostic value, especially in female patients.

**Table 3:** Variant allele frequencies of *SLCO2A1* gene (NM_005630.3) in patients with PHO & CEAS of different genders.

Variants(c.)	Protein change(p.)	Mutant allele frequency		
		Total (n=54)	Male (n=41)	Female (n=13)
c.764G>A	p.Gly255Glu	1 (0.93%)	1 (1.22%)	0 (0%)
c.1634delA	p.Asn545ThrfsTer15	1 (0.93%)	1 (1.22%)	0 (0%)
c.940 + 1G>A	p.Arg288GlyfsTer7	28 (25.93%)	18 (21.95%)	10 (38.46%)
c.1807C>T	p.Arg603Ter	27 (25.00%)	13 (15.85%)	14 (53.85%)
c.547G>A	p.Gly183Arg	5 (4.63%)	5 (6.10%)	0 (0%)
c.1106G>A	p.Gly369Asp	4 (3.70%)	4 (4.88%)	0 (0%)
c.941-1G>A	NA	2 (1.85%)	2 (2.44%)	0 (0%)
c.1771C>T	p.Arg591Ter	3 (2.78%)	3 (3.66%)	0 (0%)
c.1406C>T	p.Pro469Leu	1 (0.93%)	1 (1.22%)	0 (0%)
c.1602C>A	p.Asn534Lys	1 (0.93%)	1 (1.22%)	0 (0%)
c.611C>T	p.Ser204Leu	2 (1.85%)	2 (2.44%)	0 (0%)
c.96 + 4A>C	NA	1 (0.93%)	1 (1.22%)	0 (0%)
c.1069T>C	p.Tyr357His	1 (0.93%)	1 (1.22%)	0 (0%)
c.289C>T	p.Arg97Cys	2 (1.85%)	2 (2.44%)	0 (0%)
c.850A>G	p.Ile284Val	1 (0.93%)	1 (1.22%)	0 (0%)
c.1375T>C	p.Cys459Arg	2 (1.85%)	2 (2.44%)	0 (0%)
c.1475G>A	p.Cys492Tyr	1 (0.93%)	1 (1.22%)	0 (0%)
c.855delA	p.Ala286GlnfsTer35	4 (3.70%)	4 (4.88%)	0 (0%)
c.210G>A	p.Ser70=	1 (0.93%)	1 (1.22%)	0 (0%)
c.838C>T	p.Arg280Ter	1 (0.93%)	1 (1.22%)	0 (0%)
c.664G>A	p.Gly222Arg	4 (3.70%)	2 (2.44%)	2 (7.69%)
c.1590C>A	p.Cys530Ter	2 (1.85%)	2 (2.44%)	0 (0%)
c.1682G>A	p.Arg561His	2 (1.85%)	2 (2.44%)	0 (0%)
c.1768del	p.Arg590fsTer39	2 (1.85%)	2 (2.44%)	0 (0%)
c.929A>G	p.Asp310Gly	4 (3.70%)	4 (4.88%)	0 (0%)
c.310G>A	p.Glu104Lys	1 (0.93%)	1 (1.22%)	0 (0%)
c.547dup	NA	1 (0.93%)	1 (1.22%)	0 (0%)
c.1259G>T	p.Cys420Phe	2 (1.85%)	2 (2.44%)	0 (0%)
c.1177delT	p.Ser393LeufsTer8	1 (0.93%)	1 (1.22%)	0 (0%)

Counting rule: A homozygous mutation is counted as 2 occurrences, whereas each mutation in a compound heterozygous state is counted as 1 occurrence. The above counts are based on the genotype information of 54 patients in the table (including both homozygous and compound heterozygous mutations). Only three variants c.940 + 1G>A, c.1807C>T and c.664G>A were detected in female patients; all other mutations were exclusively identified in male patients.

**Figure 4:**
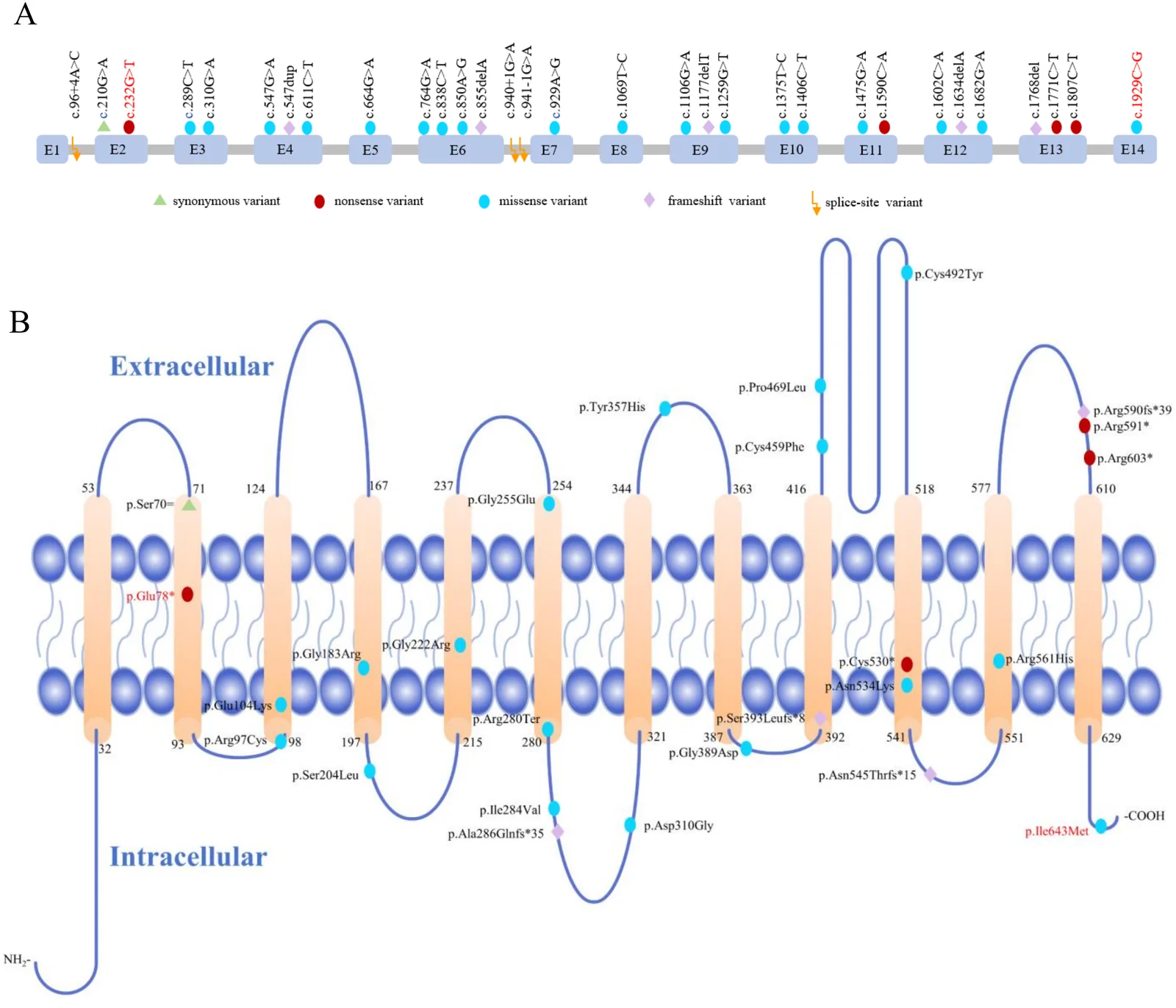
Spectrum of reported *SLCO2A1* gene variants in patients diagnosed with both PHO and CEAS **(A)** Germline pathogenic variant loci within the *SLCO2A1* gene; **(B)** The spatial distribution of amino acid substitutions in *SLCO2A1*. The light blue spheres aligned tail-to-tail symbolize the cell membrane; the region above the membrane indicates the extracellular side, and the region below indicates the intracellular side. The orange bars illustrate the 12 transmembrane domains of *SLCO2A1*, and the thin blue connecting lines indicate the intracellular and extracellular regions of the protein. The numbers mark the start and end amino acid residue positions of each transmembrane domain. -NH_2_ and -COOH refer to the N‑terminus and C‑terminus, respectively. The variants identified in the sibling are highlighted in red font. PHO, primary hypertrophic osteoarthropathy; CEAS, chronic enteropathy associated with the *SLCO2A1* gene; E1-E14, exons 1 to 14 of *SLCO2A1* gene.

To discuss the relationship between genotype and phenotype, we analyzed the domains or regions in which the variants were located. The human *SLCO2A1* gene is located on chromosome 3q22.1-q22.2 and comprises 14 exons (**Figure 4A**). The protein encoded by *SLCO2A1* consists of 643 amino acids and includes 12 transmembrane domains (TM). The distribution of the 29 identified variations across protein structural domains is relatively homogeneous (**Figure 4B**). After excluding truncating or splice-site variants, 10 of the remaining 17 variants mapped directly to the SLCO2A1-PGE2 binding site. These involve TM5, TM7, TM8, TM10 and TM11, the TM6-TM7 amphipathic cytoplasmic helix, and the TM9-TM10 extracellular loop.

## 4 Discussion

The *SLCO2A1* gene encodes solute carrier organic anion transporter family member 2A1, a prostaglandin transporter (PGT) featuring twelve transmembrane domains. Defective *SLCO2A1* function has been directly associated with two rare monogenic disorders: PHO and CEAS. The cases of PHO and CEAS were first reported in 1868 and 2015, respectively, whereas cases presenting with both PHO and CEAS were described more recently. In this study, we reported two Chinese patients with both PHO and possible CEAS and identified two novel compound heterozygous variants in *SLCO2A1*. The nonsense variant NM_005630.3: c.232G>T (p.Glu78Ter) introduces a premature stop codon at position 78 within the membrane helix, resulting in the truncation of 565 amino acids, which might trigger nonsense-mediated mRNA degradation and lead to premature peptide chain termination. The missense variant NM_005630.3: c.1929C>G (p.Ile643Met) leads to the substitution of isoleucine for methionine at position 643. Although *in silico* prediction algorithms have classified this variant as “likely pathogenic”, it remains essential to perform variant-specific protein functional assays. This is particularly critical for applying the PS3 (well-established *in vitro* or *in vivo* functional studies supporting a damaging effect on the gene or gene product) evidence criteria. A recent study reported that 6-Carboxyfluorescein (6-CF) can be internalized by the SLCO2A1 transporter and has been effectively used to assess the impact of *SLCO2A1* variants. In the study, the variants p.Arg97Cys, p.Gly183Arg, p.Gly222Arg, and p.Cys492Tyr were shown to nearly abolish 6-CF uptake (11). The lack of independent *in vitro* functional assays for these variants represents a limitation of the current work.

Many clinical manifestations of CEAS overlap with those of other intestinal disorders, such as Crohn’s disease, Behcet’s disease, and simple ulcers. Although glucocorticoids and thiopurines are widely used to treat Crohn’s disease and ulcerative colitis, they have not demonstrated efficacy in treating CEAS (20, 21). Consequently, it is imperative to accurately differentiate CEAS from other inflammatory bowel diseases. Clinical investigations have revealed that urinary concentrations of the major prostaglandin E metabolite (PGE-M) are significantly elevated in patients with CEAS compared to those with other bowel diseases, indicating that PGE-M is a valuable diagnostic biomarker for distinguishing CEAS (14, 22). Furthermore, immunohistochemical staining for *SLCO2A1* in gastroduodenal tissues may serve as an effective alternative method for differentiating CEAS from other bowel disorders (23, 24). In this study, although both siblings were diagnosed with concurrent PHO and possible CEAS, the absence of diagnostic procedures such as capsule endoscopy, balloon-assisted enteroscopy, and specialized small-bowel imaging resulted in the failure to identify the characteristic lesions in the small intestine. This represents an additional limitation of the study.

While no definitive cure exists for PHO and CEAS, symptomatic treatment remains the primary therapeutic approach. It is now widely recognized that etoricoxib is the preferred selective COX-2 inhibitor for managing PHO and CEAS (25, 26), with studies demonstrating its safety and efficacy (27, 28). In this study, patients receiving a combination therapy of etoricoxib, colchicine, and topical isotretinoin showed a notable reduction in facial skin induration and a significant alleviation of knee swelling. However, research has also demonstrated that PGE2 exerts mucosal-protective effects in the small intestine by promoting epithelial cell turnover and repair and maintaining intercellular tight junctions (10). The increase in PGE_2_ levels in the intestinal mucosa can promote healing of small-bowel lesions by stimulating angiogenesis through up-regulation of VEGF expression (29). Washio E. et al. reported that 16.7% of healthy volunteers exhibited some small intestinal mucosal injuries after two-week administration of a selective COX-2 inhibitor (30). Therefore, it is crucial to consider potential side effects when treating patients with CEAS with selective COX-2 inhibitors. It is recommended that capsule endoscopy and blood tests be performed throughout the treatment period to monitor these effects (31).

There are notable differences in diagnostic challenges between PHO and CEAS, particularly in females. PHO predominantly affects males, and all patients recruited in this study presented with digital clubbing (100%), pachydermia (100%), cutis gyrata (100%), arthralgia (97.06%), and periosteal thickening (100%). These typical clinical features of PHO facilitate its diagnosis (6, 7). In contrast, CEAS has a higher prevalence among females, and all female patients in this study exhibited gastrointestinal symptoms. However, the considerable variability in these gastrointestinal symptoms makes diagnosis more challenging for females (8). Furthermore, both our research and previous studies suggested that male patients often overlook gastrointestinal symptoms, whereas female patients are commonly diagnosed based on gastrointestinal symptoms rather than CEAS. Accordingly, we propose that genetic diagnosis should be routinely performed in these cohorts.

Although the genetic characteristics of patients with PHO or CEAS have been extensively described globally, the genetic profile of *SLCO2A1* variants in individuals presenting with both PHO and CEAS remains limited (1, 18, 19). We summarized all variants in the *SLCO2A1* gene from patients with PHO and CEAS (**Figures 4A, B**) and found that the distribution of variants across protein structural domains was relatively homogeneous. Notably, the variant allele frequency (VAF) of the NM_005630.3: c.940 + 1G>A (p.Arg288GlyfsTer7) and NM_005630.3: c.1807C>T (p.Arg603Ter) variants were 25.9% and 25%, respectively. These findings are consistent with previous studies (5, 14).

**Figure 5:**
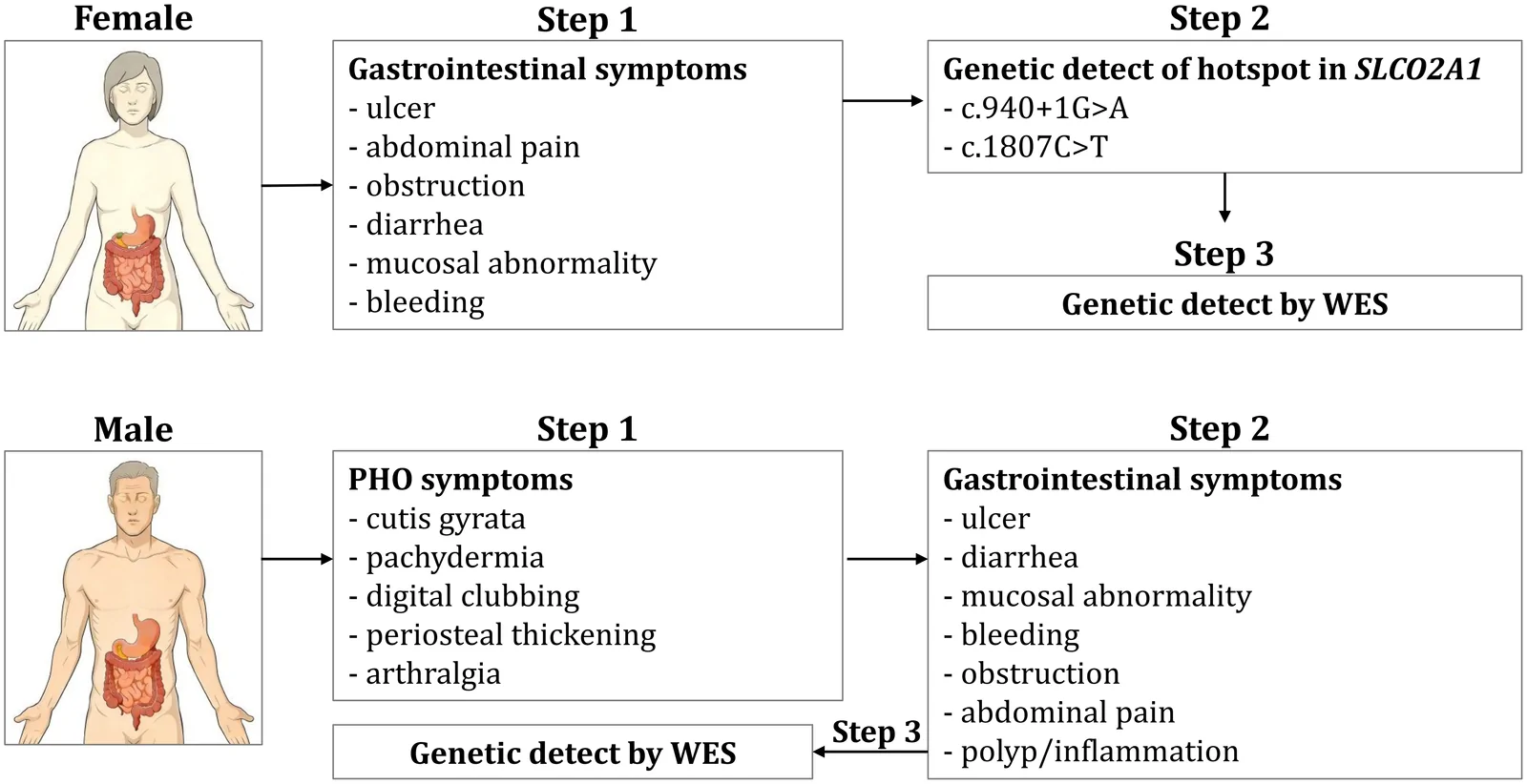
The proposed recommended clinical consideration for PHO and CEAS. In females, the proposed recommended clinical consideration involves an assessment of gastrointestinal symptoms associated with CEAS, followed by SLCO2A1 hotspot testing (c.940+1G>A and c.1807C>T), with WES available as an option if deemed necessary. In males, the proposed recommended clinical consideration includes an evaluation of clinical symptoms related to PHO, subsequent examination of gastrointestinal lesions, and concludes with genetic testing. Nonetheless, it is important to acknowledge that the proposed recommended clinical consideration must be complemented with additional confirmatory examinations: endoscopy for CEAS, and imaging modalities such as radiography and/or scintigraphy for PHO. PHO, primary hypertrophic osteoarthropathy; CEAS, chronic enteropathy associated with the *SLCO2A1* gene; WES, whole exome sequencing.

Integrating clinical and genetic characteristics, we propose a recommended clinical consideration for PHO and CEAS (**Figure 5**): 1) for females, gastrointestinal lesions constitute the primary diagnostic indicator. Next, genetic diagnosis may be performed by examining the two variant hotspots, NM_005630.3: c.940+1G>A and c.1807C>T, within the *SLCO2A1* gene. If required, additional whole-exome sequencing can be undertaken; 2) for males, the diagnostic process begins with an assessment of the clinical symptoms associated with PHO, followed by concludes with an evaluation of gastrointestinal lesions, and genetic testing. Nonetheless, it is important to acknowledge that this clinical consideration still requires further validation due to the relatively limited number of cases and the inherent limitations of the data derived from literatures.

In conclusion, this study reported two patients presenting with both PHO and possible CEAS, in whom two novel compound heterozygous variants (NM_005630.3: c.232G>T and c.1929C>G) in the *SLCO2A1* gene were identified. This expanded the spectrum of *SLCO2A1* variants. Furthermore, an analysis of the clinical and genetic characteristics of patients with concurrent PHO and CEAS was performed. Based on these findings, a recommended clinical consideration for male and female patients were proposed to enhance the diagnosis and treatment of disorders associated with *SLCO2A1* variants.

## Data Availability

All data and materials are included within the main text or **Supplementary Materials**. The whole exome sequencing data reported in this paper have been deposited in the Genome Variation Map at the National Genomics Data Center, China National Center for Bioinformation / Beijing Institute of Genomics, Chinese Academy of Sciences. These data are available under accession number GVM001494 and can be accessed upon reasonable request at the following URL: https://bigd.big.ac.cn/gvm/getProjectDetail?Project=GVM001494.
