# Supplementary material for: Biallelic SLCO2A1 variants in two siblings with primary hypertrophic osteoarthropathy and possible chronic enteropathy
Source: Front Endocrinol (Lausanne). 2026 Sep 10;17:1783445. doi: 10.3389/fendo.2026.1783445 (PMC13600845; doi:10.3389/fendo.2026.1783445)
Supplement: Supplementary file 1 [file Table1.doc]

**SUPPLEMENTARY TABLE 1** The criteria of variant classification used in this study

| **Variant** | **ACMG criteria applied** |  |
| --- | --- | --- |
| ***SLCO2A1***  **NM_005630.3: c.232G>T(p.E78*)** | PVS1 | Null variant (nonsense) in a gene where LOF is a known mechanism of disease |
|  | PM2_Supporting | Absent from controls in Genome Aggregation Database. |
|  | PP1 | Co-segregation with disease |
|  | PP4 | Patient's phenotype is highly specific for a disease with a single genetic etiology. |
| ***SLCO2A1***  **NM_005630.3: c.1929C>G (p.I643M)** | PM2_Supporting | Absent from controls in Genome Aggregation Database. |
|  | PM3 | For recessive disorders, detected in trans with a pathogenic variant. |
|  | PP1 | Co-segregation with disease |
|  | PP3 | Computational evidence (PolyPhen-2 and SIFT) supports a deleterious effect on the gene or gene product. |
|  | PP4 | Patient's phenotype is highly specific for a disease with a single genetic etiology. |
