# Supplementary material for: Biallelic SLCO2A1 variants in two siblings with primary hypertrophic osteoarthropathy and possible chronic enteropathy
Source: Front Endocrinol (Lausanne). 2026 Sep 10;17:1783445. doi: 10.3389/fendo.2026.1783445 (PMC13600845; doi:10.3389/fendo.2026.1783445)
Supplement: Supplementary file 2 [file Table2.doc]

**SUPPLEMENTARY TABLE 2** Clinical and genetic characteristics of patients diagnosed with both PHO and CEAS

| References | Patient | Gender | Onset age | Gastrointestinal symptoms | Cutis gyrate | Pachydermia | Digital clubbing | Periosteal thickening | Arthralgia | Variants in *SLCO2A1* | Mutation type | Protein change |
| --- | --- | --- | --- | --- | --- | --- | --- | --- | --- | --- | --- | --- |
| 22197487(1) | P1 | Male | NA | Gastric ulcer, Gastric bleeding | + | + | + | NA | NA | c.764G>A | Compound heterozygous | p.Gly255Glu |
|  |  |  |  |  |  |  |  |  |  | c.1634delA |  | p.Asn545ThrfsTer15 |
| 24012041(2) | P1 | Male | 16 | Mild gastric hypertrophy | + | + | + | + | + | c.940+1G>A | Homozygous | p.Arg288GlyfsTer7 |
| 24153155(3) | P1 | Male | 20 | Gastric polyps, Erosive gastritis | + | + | + | + | + | c.1807C>T | Compound heterozygous | p.Arg603Ter |
|  |  |  |  |  |  |  |  |  |  | c.547G>A |  | p.Gly183Arg |
| 25810087(4) | P1 | Male | 13 | Watery diarrhea | + | + | + | + | + | c.940+1G>A | Homozygous | p.Arg288GlyfsTer7 |
|  | P2 | Male | NA | Watery diarrhea | + | + | + | + | + | c.940+1G>A | Homozygous | p.Arg288GlyfsTer7 |
| 28851954(5) | P1 | Male | 19 | Gastric mucosal hyperplasia | + | + | + | + | + | c.1106G>A | Homozygous | p.Gly369Asp |
|  | P3 | Male | 21 | Gastric mucosal hyperplasia | + | + | + | + | + | c.1106G>A | Compound heterozygous | p.Gly369Asp |
|  |  |  |  |  |  |  |  |  |  | c.1807C>T |  | p.Arg603Ter |
|  | P4 | Male | 15 | Gastric mucosal hyperplasia | + | + | + | + | + | c.941-1G>A | Compound heterozygous | NA |
|  |  |  |  |  |  |  |  |  |  | c.1771C>T |  | p.Arg591Ter |
|  | P5 | Male | 19 | Gastric mucosal hyperplasia | + | + | + | + | + | c.1406C>T | Compound heterozygous | p.Pro469Leu |
|  |  |  |  |  |  |  |  |  |  | c.1602C>A |  | p.Asn534Lys |
|  | P6 | Male | 14 | Gastric mucosal hyperplasia | + | + | + | + | + | c.611C>T | Homozygous | p.Ser204Leu |
|  | P7 | Male | 16 | Gastric mucosal hyperplasia | + | + | + | + | + | c.96+4A>C | Compound heterozygous | NA |
|  |  |  |  |  |  |  |  |  |  | c.1069T>C |  | p.Tyr357His |
| 28916395(6) | P1 | Male | 31 | Gastric multiple ulcers, Small intestinal multiple ulcers | + | + | + | + | + | c.289C>T | Homozygous | p.Arg97Cys |
| 29239930(7) | P1 | Male | 17 | Watery diarrhea | + | + | + | + | + | c.547G>A | Homozygous | p.Gly183Arg |
| 30880718(8) | P1 | Male | 15 | Gastric ulcer, Gastric mucosal hyperplasia, Hematemesis | + | + | + | + | + | c.1807C>T | Homozygous | p.Arg603Ter |
| 31004291(9) | P1 | Male | 16 | Watery diarrhea | NA | + | + | + | + | c.850A>G | Compound heterozygous | p.Ile284Val |
|  |  |  |  |  |  |  |  |  |  | c.1375T>C |  | p.Cys459Arg |
| 31611528(10) | P1 | Male | 30 | Gastric multiple ulcers, Small intestinal multiple ulcers, Anemia | + | + | + | + | NA | c.940+1G>A | Compound heterozygous | p.Arg288GlyfsTer7 |
|  |  |  |  |  |  |  |  |  |  | c.1475G>A |  | p.Cys492Tyr |
| 31878983(11) | P1 | Male | 18 | Intestinal obstruction Diarrhea | NA | + | + | + | + | c.1807C>T | Homozygous  Nonsense | p.Arg603Ter |
|  | P2 | Male | 22 | Diarrhea, Abdominal pain, Anemia | NA | + | + | + | + | c.855delA | Homozygous | p.Ala286GlnfsTer35 |
| 33308032(12) | P1 | Male | 12 | Gastric ulcer, Intestinal obstruction | + | + | + | + | + | c.940+1G>A | Homozygous | p.Arg288GlyfsTer7 |
| 33328413(13) | P1 | Male | 46 | Gastric ulcers, Ileal ulcers | + | + | + | NA | NA | c.1807C>T | Homozygous | p.Arg603Ter |
| 34052181(14) | P1 | Male | NA | Small intestinal multiple ulcers, Anemia | NA | + | + | + | - | c.210G>A | Compound heterozygous | p.Ser70= |
|  |  |  |  |  |  |  |  |  |  | c.838C>T |  | p.Arg280Ter |
| 34114674(15) | P1 | Male | NA | Diarrhea | + | + | + | + | NA | c.940+1G>A | Homozygous  splice-site | p.Arg288GlyfsTer7 |
|  | P2 | Male | 15 | Duodenal ulcer | NA | + | + | + | NA | c.940+1G>A | Homozygous | p.Arg288GlyfsTer7 |
|  | P3 | Male | 15 | Chronic diarrhea | + | + | + | + | NA | c.664G>A | Homozygous | p.Gly222Arg |
|  | P4 | Male | 17 | Melena | + | + | + | + | NA | c.940+1G>A | Compound heterozygous | p.Arg288GlyfsTer7 |
|  |  |  |  |  |  |  |  |  |  | c.1807C>T |  | p.Arg603Ter |
| 35611666(16) | P1 | Female | 21 | Jejunal ulcers, Ileal ulcers | - | - | + | + | - | c.940+1G>A | Homozygous | p.Arg288GlyfsTer7 |
|  | P2 | Female | 32 | Ileal stenosis | - | - | + | NA | + | c.940+1G>A | Compound heterozygous | p.Arg288GlyfsTer7 |
|  |  |  |  |  |  |  |  |  |  | c.1807C>T |  | p.Arg603Ter |
|  | P3 | Female | 37 | Gastric ulcers, Duodenal ulcers, Ileal ulcers, Colonic ulcers | - | - | - | NA | - | c.1807C>T | Homozygous | p.Arg603Ter |
|  | P4 | Female | 34 | Gastric ulcers, Duodenal ulcers, Ileal ulcers | - | - | - | NA | - | c.1807C>T | Homozygous | p.Arg603Ter |
|  | P5 | Female | 16 | Ileal multiple ulcers | - | - | - | NA | - | c.940+1G>A | Compound heterozygous | p.Arg288GlyfsTer7 |
|  |  |  |  |  |  |  |  |  |  | c.1807C>T |  | p.Arg603Ter |
|  | P6 | Male | 11 | Duodenal ulcers, Jejunal ulcers, Ileal ulcers, Colonic ulcers | NA | + | + | + | + | c.940+1G>A | Compound heterozygous | p.Arg288GlyfsTer7 |
|  |  |  |  |  |  |  |  |  |  | c.1807C>T |  | p.Arg603Ter |
|  | P7 | Female | 12 | Ileal ulcers | - | - | - | NA | - | c.940+1G>A | Compound heterozygous | p.Arg288GlyfsTer7 |
|  |  |  |  |  |  |  |  |  |  | c.1807C>T |  | p.Arg603Ter |
|  | P8 | Female | 11 | Jejunal ulcers, Ileal ulcers | - | - | - | NA | - | c.940+1G>A | Compound heterozygous | p.Arg288GlyfsTer7 |
|  |  |  |  |  |  |  |  |  |  | c.1807C>T |  | p.Arg603Ter |
|  | P9 | Female | 12 | Ileal thickening | - | - | - | NA | - | c.940+1G>A | Compound heterozygous | p.Arg288GlyfsTer7 |
|  |  |  |  |  |  |  |  |  |  | c.1807C>T |  | p.Arg603Ter |
|  | P10 | Female | NA | Ileal ulcers | NA | + | + | + | - | c.1807C>T | Homozygous | p.Arg603Ter |
|  | P11 | Female | NA | Jejunal ulcers, Ileal ulcers | - | - | - | NA | - | c.940+1G>A | Compound heterozygous | p.Arg288GlyfsTer7 |
|  |  |  |  |  |  |  |  |  |  | c.1807C>T |  | p.Arg603Ter |
|  | P12 | Female | NA | Duodenal ulcers, jejunal ulcers, ileal ulcers | - | - | - | NA | - | c.940+1G>A | Homozygous | p.Arg288GlyfsTer7 |
|  | P13 | Male | 15 | Duodenal stenosis, Jejunal stenosis, Ileal stenosis | NA | + | + | + | + | c.940+1G>A | Compound heterozygous | p.Arg288GlyfsTer7 |
|  |  |  |  |  |  |  |  |  |  | c.1807C>T |  | p.Arg603Ter |
|  | P14 | Female | NA | Ileal ulcers | - | - | - | NA | - | c.1807C>T | Homozygous | p.Arg603Ter |
| 36089247(17) | PP1 | Female | 15 | Chronic diarrhea | - | - | + | + | - | c.664G>A | Homozygous | p.Gly222Asp |
| 36549465(18) | P4 | Male | 5 | Chronic diarrhea | + | + | + | + | + | c.1590C>A | Homozygous | p.Cys530Ter |
|  | P6 | Male | 20 | Malabsorption | + | + | + | + | + | c.1682G>A | Homozygous | p.Arg561His |
| 37915296(19) | P1 | Male | 12 | Duodenal Multiple ulcers, Hemorrhage | + | + | + | + | + | c.547G>A | Homozygous | p.Gly183Arg |
|  | P2 | Male | 15 | Congestive gastropathy | + | + | + | + | + | c.1768del | Homozygous | p.Arg590fsTer39 |
| 37991153(20) | P1 | Male | 14 | Diarrhea | + | + | + | + | + | c.940+1G>A | Homozygous | p.Arg288GlyfsTer7 |
| 38862970(21) | P1 | Male | 22 | Sigmoid diffuse mucosal edema, Rectal diffuse mucosal edema | + | + | + | + | + | c.929A>G | Homozygous | p.Asp310Gly |
| 39489190(22) | P1 | Male | 12 | Gastric multiple polyps, Gastric mucosal hypertrophy | + | + | + | + | + | c.310G>A | Compound heterozygous | p.Glu104Lys |
|  |  |  |  |  |  |  |  |  |  | c.547dup |  | NA |
| 40144454(23) | P2 | Male | 17 | Diarrhea | + | + | + | + | + | c.1259G>T | Homozygous | p.Cys420Phe |
| 10.3760/cma.j.issn.1008-1372.2020.01.043(24) | P1 | Male | 16 | Diarrhea, Gastric hemorrhage | NA | + | + | + | + | c.1771C>T | Homozygous | p.Arg591Ter |
| 10.3760/cma.j.cn112138-20200318-00259(25) | P1 | Male | 16 | Intestinal obstruction, Abdominal pain, Diarrhea | NA | + | + | + | + | c.1807C>T | Homozygous | p.Arg603Ter |
|  | P2 | Male | 14 | Abdominal pain, Diarrhea | NA | + | + | + | + | c.855delA | Homozygous | p.Ala286GlnfsTer35 |
|  | P3 | Male | 22 | Intestinal obstruction, Abdominal pain, Diarrhea | NA | + | + | + | + | c.929A>G | Homozygous | p.Asp310Gly |
|  | P4 | Male | 16 | Intestinal obstruction, Abdominal pain, Diarrhea | NA | + | + | + | + | c.941-1G>A | Compound heterozygous | NA |
|  |  |  |  |  |  |  |  |  |  | c.1106G>A |  | p.Gly369Asp |
|  | P5 | Male | 23 | Intestinal obstruction, Abdominal pain, Diarrhea | NA | + | + | + | + | c.1177delT | Compound heterozygous | p.Ser393LeufsTer8 |
|  |  |  |  |  |  |  |  |  |  | c.1375T>C |  | p.Cys459Arg |

Onset age: years; NA: no information; +: positive; -: negative.

**REFERENCE**

1.	Zhang Z, Xia W, He J, Zhang Z, Ke Y, Yue H, et al. Exome Sequencing Identifies Slco2a1 Mutations as a Cause of Primary Hypertrophic Osteoarthropathy. *American journal of human genetics* (2012) 90(1):125-32. Epub 2011/12/27. doi: 10.1016/j.ajhg.2011.11.019.

2.	Madruga Dias JA, Rosa RS, Perpétuo I, Rodrigues AM, Janeiro A, Costa MM, et al. Pachydermoperiostosis in an African Patient Caused by a Chinese/Japanese Slco2a1 Mutation-Case Report and Review of Literature. *Seminars in arthritis and rheumatism* (2014) 43(4):566-9. Epub 2013/09/10. doi: 10.1016/j.semarthrit.2013.07.015.

3.	Cheng R, Li M, Guo Y, Yao Y, Gao C, Yao Z. Three Novel Mutations in the Slco2a1 Gene in Two Chinese Families with Primary Hypertrophic Osteoarthropathy. *European journal of dermatology : EJD* (2013) 23(5):636-9. Epub 2013/10/25. doi: 10.1684/ejd.2013.2154.

4.	Kim HJ, Koo KY, Shin DY, Kim DY, Lee JS, Lee MG. Complete Form of Pachydermoperiostosis with Slco2a1 Gene Mutation in a Korean Family. *The Journal of dermatology* (2015) 42(6):655-7. Epub 2015/03/27. doi: 10.1111/1346-8138.12856.

5.	Huang H, Wang Y, Cao Y, Wu B, Li Y, Fan L, et al. Interleukin-6, Tumor Necrosis Factor-Alpha and Receptor Activator of Nuclear Factor Kappa Ligand Are Elevated in Hypertrophic Gastric Mucosa of Pachydermoperiostosis. *Scientific reports* (2017) 7(1):9686. Epub 2017/08/31. doi: 10.1038/s41598-017-09671-7.

6.	Seta V, Capri Y, Battistella M, Bagot M, Bourrat E. Pachydermoperiostosis: The Value of Molecular Diagnosis. *Annales de dermatologie et de venereologie* (2017) 144(12):799-803. Epub 2017/09/17. doi: 10.1016/j.annder.2017.03.027.

7.	Sun F, Guo L, Ye S. Primary Hypertrophic Osteoarthropathy with Slco2a1 Mutation in a Chinese Patient Successfully Treated with Etoricoxib. *Journal of clinical rheumatology : practical reports on rheumatic & musculoskeletal diseases* (2018) 24(3):164-7. Epub 2017/12/15. doi: 10.1097/rhu.0000000000000647.

8.	Li X, Hao D, Li-Ling J, Jiang X. Complete Form of Pachydermoperiostosis with Cutis Verticis Gyrata Resulting from the Slco2a1 Gene Mutation. *Indian journal of dermatology, venereology and leprology* (2019) 85(6):681. Epub 2019/03/19. doi: 10.4103/ijdvl.IJDVL_911_17.

9.	Jiang Y, Du J, Song YW, Wang WB, Pang QQ, Li M, et al. Novel Slco2a1compound Heterozygous Mutation Causing Primary Hypertrophic Osteoarthropathy with Bartter-Like Hypokalemia in a Chinese Family. *Journal of endocrinological investigation* (2019) 42(10):1245-52. Epub 2019/04/21. doi: 10.1007/s40618-019-01048-z.

10.	Sonoda A, Wada Y, Togo K, Mizukami K, Fuyuno Y, Umeno J, et al. Characteristic Facial Appearance Was the Key to Diagnosing Chronic Enteropathy Associated with Slco2a1-Associated Primary Hypertrophic Osteoarthropathy. *Internal medicine (Tokyo, Japan)* (2020) 59(4):491-4. Epub 2019/10/16. doi: 10.2169/internalmedicine.3369-19.

11.	Wang Q, Li YH, Lin GL, Li Y, Zhou WX, Qian JM, et al. Primary Hypertrophic Osteoarthropathy Related Gastrointestinal Complication Has Distinctive Clinical and Pathological Characteristics: Two Cases Report and Review of the Literature. *Orphanet journal of rare diseases* (2019) 14(1):297. Epub 2019/12/28. doi: 10.1186/s13023-019-1264-5.

12.	Ishizuka T, Fujioka K, Mori I, Takeda T, Fuwa M, Ikeda T, et al. Primary Hypertrophic Osteoarthropathy with Severe Arthralgia Identified by Gene Mutation of Slco2a1. *Modern rheumatology case reports* (2021) 5(2):404-8. Epub 2020/12/15. doi: 10.1080/24725625.2020.1861744.

13.	Tsuzuki Y, Aoyagi R, Miyaguchi K, Ashitani K, Ohgo H, Yamaoka M, et al. Chronic Enteropathy Associated with Slco2a1 with Pachydermoperiostosis. *Internal medicine (Tokyo, Japan)* (2020) 59(24):3147-54. Epub 2020/12/18. doi: 10.2169/internalmedicine.4756-20.

14.	Sun K, He Q, Zhu L, Abula G, Zhao J, Chen X. Multiple Small Intestinal Ulcers with Slco2a1 and Pla2g4a Mutation in a Chinese Patient. *Digestive and liver disease : official journal of the Italian Society of Gastroenterology and the Italian Association for the Study of the Liver* (2021) 53(8):1062-4. Epub 2021/05/31. doi: 10.1016/j.dld.2021.04.032.

15.	Oiwa T, Ishibashi M, Okuno T, Ohba M, Endo Y, Uozumi R, et al. Eicosanoid Profiling in Patients with Complete Form of Pachydermoperiostosis Carrying Slco2a1 Mutations. *The Journal of dermatology* (2021) 48(9):1442-6. Epub 2021/06/12. doi: 10.1111/1346-8138.16012.

16.	Hong HS, Baek J, Park JC, Lee HS, Park D, Yoon AR, et al. Clinical and Genetic Characteristics of Korean Patients Diagnosed with Chronic Enteropathy Associated with Slco2a1 Gene: A Kasid Multicenter Study. *Gut and liver* (2022) 16(6):942-51. Epub 2022/05/26. doi: 10.5009/gnl210415.

17.	Long B, Tang H, Zhao X, He T, Tang M, Wan P. Chronic Enteropathy Associated with Slco2a1-Associated Primary Hypertrophic Osteoarthropathy in a Female Patient. *Clinics and research in hepatology and gastroenterology* (2022) 46(9):102021. Epub 2022/09/12. doi: 10.1016/j.clinre.2022.102021.

18.	Bloch A, Couture G, Isidor B, Ricquebourg M, Bourrat E, Lipsker D, et al. Novel Pathogenic Variants in Slco2a1 Causing Autosomal Dominant Primary Hypertrophic Osteoarthropathy. *European journal of medical genetics* (2023) 66(2):104689. Epub 2022/12/23. doi: 10.1016/j.ejmg.2022.104689.

19.	Kimball TN, Rivero-García P, Barrera-Godínez A, Domínguez-Cherit J. Genotype and Phenotype Characterization of Primary Hypertrophic Osteoarthropathy Type 2 and Chronic Enteropathy Associated with Slco2a1: Report of Two Cases and Literature Review. *American journal of medical genetics Part A* (2024) 194(3):e63446. Epub 2023/11/02. doi: 10.1002/ajmg.a.63446.

20.	Long B, Jiang C, Zheng Q, Wan P. Pachydermoperiostosis Combined with Pyloric Gland Adenoma with Foveolar-Type Adenoma. *United European gastroenterology journal* (2024) 12(1):152-4. Epub 2023/11/22. doi: 10.1002/ueg2.12484.

21.	Dai Y, He M, Xu H, Tan B, Zhou W, Liu W, et al. A Novel Variant in the Slco2a1 Gene in a Chinese Patient with Chronic Gastroenteropathy and Primary Hypertrophic Osteoarthropathy. *Orphanet journal of rare diseases* (2024) 19(1):229. Epub 2024/06/12. doi: 10.1186/s13023-024-03221-x.

22.	Jin H, Chen F, Yang J. An Unusual Case of Gastric Multiple Lesions with Skin and Joint Abnormalities. *Gastroenterology* (2025) 168(5):879-84. Epub 2024/11/04. doi: 10.1053/j.gastro.2024.10.031.

23.	Arcanjo AM, de Souza AF, de Souza Quedas EP, de Menezes Correia-Deur JE, Ferreira DL, de Almeida Toledo SP, et al. Primary Hypertrophic Osteoarthropathy: Phenotypic Variability and Penetrance Rate in Heterozygotes for Slco2a1 Variants. *JBMR plus* (2025) 9(4):ziaf026. Epub 2025/03/27. doi: 10.1093/jbmrpl/ziaf026.

24.	Ruimei Jiang, Juan Li, Min Niu, et al. Two cases of primary hypertrophic osteoarthropathy caused by SLCO2A1 gene abnormality in the same family. Journal of Chinese Physician J 2020; 22(01): 146-149.

25.	Wang Q, Xu H, Li Y, Liu YP, Wu D, Zhou WX, et al. [Clinical and Genetic Characteristics of Patients with Chronic Enteropathy Associated with Slco2a1 Gene]. Zhonghua nei ke za zhi (2021) 60(1):45-50. Epub 2021/01/06. doi: 10.3760/cma.j.cn112138-20200318-00259.
